# Mechanisms of Action of (Meth)acrylates in Hemolytic Activity, *in Vivo* Toxicity and Dipalmitoylphosphatidylcholine (DPPC) Liposomes Determined Using NMR Spectroscopy

**DOI:** 10.3390/ijms13010758

**Published:** 2012-01-12

**Authors:** Seiichiro Fujisawa, Yoshinori Kadoma

**Affiliations:** 1 Meikai University School of Dentistry, Sakado, Saitama 350-0283, Japan; E-Mail: fujisawa33@nifty.com; 2 Institute of Biomaterials and Bioengineering, Tokyo Medical and Dental University, Kanda-surugadai, Chiyoda-ku, Tokyo 101-0062, Japan

**Keywords:** (meth)acrylates, QSA(P)R, biological activity, NMR, chemical shift, β-carbon, theoretical parameters, DSC, liposomes

## Abstract

We investigated the quantitative structure-activity relationships between hemolytic activity (log 1/H_50_) or *in vivo* mouse intraperitoneal (ip) LD_50_ using reported data for α,β-unsaturated carbonyl compounds such as (meth)acrylate monomers and their ^13^C-NMR β-carbon chemical shift (δ). The log 1/H_50_ value for methacrylates was linearly correlated with the δC_β_ value. That for (meth)acrylates was linearly correlated with log *P*, an index of lipophilicity. The ipLD_50_ for (meth)acrylates was linearly correlated with δC_β_ but not with log *P*. For (meth)acrylates, the δC_β_ value, which is dependent on the π-electron density on the β-carbon, was linearly correlated with PM3-based theoretical parameters (chemical hardness, *η*; electronegativity, *χ*; electrophilicity, *ω*), whereas log *P* was linearly correlated with heat of formation (HF). Also, the interaction between (meth)acrylates and DPPC liposomes in cell membrane molecular models was investigated using ^1^H-NMR spectroscopy and differential scanning calorimetry (DSC). The log 1/H_50_ value was related to the difference in chemical shift (ΔδHa) (Ha: H (*trans*) attached to the β-carbon) between the free monomer and the DPPC liposome-bound monomer. Monomer-induced DSC phase transition properties were related to HF for monomers. NMR chemical shifts may represent a valuable parameter for investigating the biological mechanisms of action of (meth)acrylates.

## 1. Introduction

**Figure 1:**
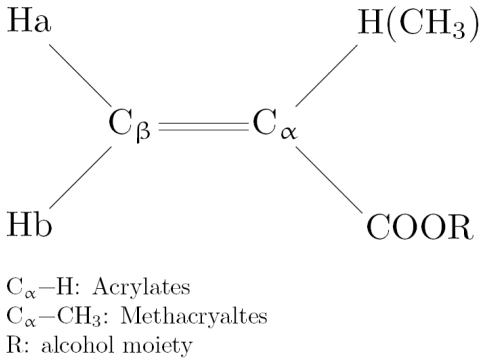
The structure of acrylates and methacrylates.

Acrylates and methacrylates, (meth)acrylates (Figure 1), are widely used in the formulation of polymeric materials for medical, dental and industrial applications.

There have been many reports on the local and systemic toxicities of these monomers [1–5], whose volatility makes them a potential hazard in working environments [6]. In particular, methacrylates are widely used in resinous dental materials for dentures and cements, and also for restorations [7]. Methyl methacrylate (MMA) has been used as a bone cement for the fixation of prosthetic joints to adjacent bone [8]. Acrylates and methacrylates generally do not polymerize completely in air because oxygen acts as a biradical and suppresses polymerization. Residual unpolymerized free monomers on resin surfaces are released into bio-systems through direct contact or inhalation. Therefore, the potential toxicity of these monomers is a primary concern when developing new medical and dental materials. Dillingham *et al.* [3] and Tanii and Hashimoto [2] previously reported quantitative structure-activity relationships (QSARs) between the *in vivo* lethal dose (LD_50_) and the lipophilicity factor (log *P*) for acrylates and methacrylates based on the Hansch model [9]. The former researchers reported a good QSAR in terms of both log *P* and the molar refraction (MR), whereas the latter reported a good QSAR in terms of both log *P* and glutathione (GSH) reactivity. Dillingham *et al*. [3] also investigated the relationships between the hemolytic activity and ipLD_50_ of (meth)acrylates and found that the mechanism responsible for the toxicity of acrylates and methacrylates was membrane-mediated and relatively non-specific, and that *in vivo* biotransformation was not a significant factor for acute toxicity. However, the biotransformation of active acrylates has been reported to be causally linked to toxicity *in vivo* [10]. Freidig *et al*. [11] previously investigated the alkaline-catalyzed hydrolysis rate constants and GSH reactivity for (meth)acrylates and found that the hydrolysis rate constants and GSH reactivity of monomers are involved in their toxicity. Chan and O’Brien [5] investigated QSARs in terms of GSH reactivity, lowest unoccupied molecular orbital (LUMO), or the partial charge on the C_β_, C_α_ and carbonyl carbons for the experimentally determined hepatocyte IC_50_, and reported rat LD_50_ data for (meth)acrylates, demonstrating a correlation between *in vitro* or *in vivo* toxicity and physico-chemical parameters for separation of acrylates and methacrylates.

According to QSAR models used by the U.S. Environmental Protection Agency (EPA) for screening information data set (SIDS) endpoints, the chemical and physical properties of methacrylates, including their melting point, boiling point, vapor pressure, log *P* (*K*_ow_) and water solubility, are strongly independent variables, and Log *P* in the Hansch model has been employed successfully as an independent variable in QSAR equations [12]. Eroglu *et al.* have employed quantum-mechanical-based descriptors in quantitative structure-toxicity relationship (QSTR) equations for organic compounds using the AM1, PM3, and DFT levels of the theory [13].

To interpret the molecular mechanism responsible for the hemolytic activity of methacrylates, we previously investigated QSARs using the PM3-based theoretical parameters for monomers and found that the theoretical data were useful for estimating the mechanisms of hemolytic activity and toxicity [14,15]. Putz *et al*. have recently investigated QSARs for organic toxicants using chemical hardness (*η*) and electronegativity (*χ*) principles in addition to Hansch indices, and found that the mechanism responsible for the genotoxic carcinogenesis of toxicants can be interpreted using computational chemistry [16]. The highest occupied molecular orbital (HOMO) and LUMO for (meth)acrylates are evident at their α,β-unsaturated carbons [17]. The ^13^C-NMR chemical shift of the β-carbon (δC_β_) of monomers is also quantitatively related to the π-electron density. The higher the π-electron density on the β-carbon, the higher the magnetic field where the NMR peak is observed; that is, as the π-electron density increases, the chemical shift value (δ) becomes smaller. Hence, it would be reasonable to correlate the magnitude of the chemical shift with the reactivity of acrylates and methacrylates [18]. Apart from log *P*, we have previously reported that the NMR chemical shifts of the α,β-unsaturated carbons for (meth)acrylates may be responsible for the toxicity resulting from reaction with tissue nucleophiles via Michael addition based on QSAR studies of published LD_50_ data in mice; there was a good correlation between the NMR chemical shifts of the β-carbon and the reactivity of reduced glutathione (GSH), and also between the GSH reactivity and LD_50_ [15,19]. However, *in vitro-in vivo* correlation studies have indicated that the 50% cytotoxic concentration (IC_50_) values for (meth)acrylates cannot be used reliably to predict LD_50_ values with a reasonable degree of precision [15].

Here, in the light of currently available data, we investigated whether the NMR chemical shifts (δHa and δC_β_) for (meth)acrylates are useful as independent variables for QSAR studies of reported data for *in vitro* hemolytic activity (50% hemolytic concentration) and *in vivo* lethal toxicity (LD_50_ in mice) [3]. Also, to clarify the mechanism responsible for the hemolytic activity of (meth)acrylates, we used DPPC liposomes as cell membrane molecular models and investigated the interaction between DPPC liposomes and (meth)acrylates using ^1^H-NMR spectroscopy and DSC.

## 2. Results and Discussion

### 2.1. Hemolytic Activity

^1^H-NMR chemical shifts are influenced by the π-electron density of the attached carbon. Ha represents the proton *trans* to the substituent, and Hb the proton *cis* to that (Figure 1). There was a good correlation between δHa and δC_β_ for monomers [18]. The ^1^H- and ^13^C-NMR chemical shift data for nine (meth)acrylates taken from the literature [18] and their biological activity (hemolytic activity, *in vivo* toxicity), physicochemical parameters and theoretical parameters, also taken from the literature [1,3,15], are shown in Tables 1, 2 and 3, respectively. We investigated QSARs between 1/H_50_ and log *P* or NMR chemical shifts (δHa, δC_β_). The QSAR for 9 monomers (MA, EA, nPA, nBA, IBA, MMA, EMA, nPMA, and nBMA) yielded good results for log *P* (*r*^2^ = 0.95) (QSAR 1), whereas there was no QSAR for δHa. Similarly, a good linear QSAR between log 1/H_50_ and heat of formation (HF) for the same data set was obtained (*r*^2^ = 0.86) (QSAR 2). Since there was a good QSPR between log *P* and HF for monomers (*r*^2^ = 0.86), the entropic and enthalpic factors could affect the overall log *P* value. Dillingham *et al*. [3] previously reported a good QSAR between the hemolytic activity and log *P* for (meth)acrylates, and also a relationship between log *P* and molar refraction (MR) (*r*^2^ = 0.70) or molecular volume (Vm) (*r*^2^ = 0.92). We investigated a relation between Vm and HF (Table 3) for 9 (meth)acrylates and it was found that the good linear relationship was obtained at *r*^2^ = 0.97; as HF increased, Vm declined. Putz *et al*. [16] described Hansch physico-chemical parameters as follows: (1) hydrophobicity (log *P*), corresponding to trans-cellular membrane diffusion and with translation motion of the molecules; (2) polarizability, accounts for the dipole perturbation and ionic interaction; and (3) optimal total energy (Etot), which contains steric information about the molecule’s 3D structure since it is given by the equilibrium conformation. We previously investigated a relationship between log *P* and van der Waals (VDW) area, dipole moment (μ) or HOMO energy for seven methacrylates monomers that were calculated using the PM3 method and it was found that two molecular parameters, VDW area and HOMO energy, particularly the former, contributed significantly to the variation of log *P* values [15]. Thus, log 1/H_50_ could be related to HF (QSAR 2).

On the other hand, a linear relationship between log 1/H_50_ and δC_β_ was obtained for separations of acrylates and methacrylates, particularly the latter (*r*^2^ = 0.87). For acrylates, there was a good linear QSAR between log 1/H_50_ and HF (*r*^2^ = 0.96) or log *P* (*r*^2^ = 0.95), but not δC_β_ (*r*^2^ = 0.46). Thus, the hemolytic mechanism may differ between acrylates and methacrylates.

Acrylates and methacrylates can be classified together as a single group of unspecifically reactive chemicals [21], or as two groups: the acrylates as electrophiles and the methacrylates as ester narcotics [22]. In general, QSARs for acrylates and methacrylates are based on the assumption that their monomers with the same functional group (e.g., R: the alcohol moiety in Figure 1) have the same mode of action. However, the classes of these chemicals may not be identical to each other because the metabolic activity or conjugation with GSH differs between acrylates and methacrylates; a difference in GSH reactivity (*k*_GSH_) has been observed between readily reactive acrylates and more slowly reactive methacrylates [5,11,20]. We previously investigated the relationship between *k*_GSH_ values using reported data and δC_β_ values for 12 acrylates and methacrylates and it was found that a good relationship was obtained [19]. The unsaturated β-carbon atom in (meth)acrylate molecules is the most probably site of attack in the Michael addition [11]. As shown in QSPR 1, in this work there was a significant relationship between GSH rate constants and δC_β_ for (meth)acrylates. McCarthy *et al.* [20] investigated depression of the erythrocyte GSH by acrylates and methacrylates *in vitro*, and found that alkylation of the erythrocyte membrane may be result from interaction between erythrocytes and the reactive acrylates, MA and EA, and also that there may be a process that can reduce the effective intracellular acrylate concentration, consequently leading to a decrease of cellular GSH depression in the erythrocyte system. Koleva *et al*. [23] reported that α,β-unsaturated carbonyl compounds such as acrylates are common environmental pollutants that are able to interact with proteins, enzymes, and DNA through various mechanisms. A common mechanism of action (Michael-type addition) may not be responsible for the hemolytic activity of acrylates because there was no QSAR between log 1/H_50_ and δC_β_ for these monomers. Therefore we investigate the hemolytic mechanism of acrylates and methacrylates using DPPC liposomes as a model of erythrocyte membranes. The results are described in Section 2.3.

### 2.2. *In Vivo* Toxicity

Next, we investigated the QSARs between ipLD_50_ and δHa or δC_β_ for (meth)acrylates, and the results are shown in Table 4. A significant linear QSAR was obtained for δHa or δC_β_ (QSARs 3 and 4; in both cases, *r*^2^ = 0.78). An increase of δHa or δC_β_ enhanced the *in vivo* toxicity. We found good QSPRs between δC_β_ and the *χ*-, *η*- or *ω*-term (in three cases, *r*^2^ = 0.99). As expected, there were also good QSARs between ipLD_50_ and the *χ*-, *η*- or *ω*- term for methacrylates (QSARs 7, 8 and 9; *r*^2^ = 0.7 − 0.8). Furthermore, there was a parabolic, not linear, QSAR (QSAR 6) for log *P* for (meth)acrylates. This finding was similar to that reported previously [1]. The QSAR in terms of both δC_β_ and log *P* yielded a better result (QSAR 5, *r*^2^ = 0.94).

Lawrence *et al.* [1] also previously reported a good QSAR between ipLD_50_ (mice) and the σ charge on the carbonyl carbon, *Q*^σ^(C) for (meth)acrylates using the Hansch model; *Q*^σ^(C) was associated with high toxicity [9]. They obtained *Q*^σ^(C), using the method of del Re [24] employing parameters that reproduce dipole moments. We also investigated the QSPR between δC_β_ and *Q*^σ^(C) for selected (meth)acrylates and obtained a good linear QSPR (*r*^2^ = 0.99). As shown for QSAR 7, the σ charge of monomer molecules was considered to play a role in the toxicity, since ester hydrolysis and nucleophilic attack are affected by the σ charge [1,11].

According to the QSARs 3 and 4, (meth)acrylates with a large δHa (δC_β_) value should have potent toxicity. Talalay *et al*. [25] previously reported that MA, acrylonitrile and acrolein were more highly active inducers of QR (NAD(P)H: (quinone-acceptor) oxidoreductase) in Hepa 1clc7 cells in comparison to MMA. MMA and acrylamide are inactive intracellular inducers of QR. The relationship between QR reactivity, GSH reactivity or *in vivo* toxicity and the NMR chemical shifts of the β-carbon for these active acrylates are summarized in Table 5.

The δHa (δC_β_) (ppm) declined in the order acrolein > acrylonitrile > MA > acrylamide > MMA. MA, acrylamide and acrolein, which are potent QR inducers, showed a large NMR chemical shift value of the β-carbon, compared to that of acrylamide; these compounds are known to be major intracellular reducing agents, and scavengers of reactive oxygen species (ROS) generated in various cellular processes [29]. Electrophilic xenobiotics such as vinyl monomers become conjugated to GSH and decrease its level within the cell [5,30]. When cellular GSH is exhausted, unscavenged ROS accumulate in cells, thus exerting toxic effects [31]. Ishikawa *et al.* [32] previously showed that MMA upregulates the expression of genes encoding phase II enzymes such as glutathione *S*-transferase and quinone oxidoreductase (NAD(P)H) in L929 cells. However, from the present result based on the NMR chemical shifts, it was assumed that such reactivity of MMA would be markedly lower than that of active acrylates such as acrolein, acrylonitrile and MA. Acrolein [33] and acrylonitrile [34] are well-known carcinogens. McCarthy *et al*. [20] reported that the carcinogenetic mechanism of acrylates may be related to alkylation of protein thiols involved in tumor promotion. Oxidative stress caused by these compounds, and the resulting oxidative damage, induce apoptosis and are involved in carcinogenesis. We predicted the GSH reactivity (*k*_app_) under cell-free conditions, and found that *k*_app_ declined in the order acrolein > acrylonitrile > MA > acrylamide > MMA. This strong decrease of GSH in hepatocytes for acrolein and acrylonitrile has been reported previously [35], and is supported by the predicted *k*_app_ data for these monomers. Also, the predicted ipLD_50_ value declined in the order acrolein > acrylonitrile > MA > acrylamide > MMA. Both acrolein and acrylonitrile were most toxic, as supported by reported oral-LD_50_ data. The induction of QR activity and GSH reactivity for active acrylates was dependent on the NMR chemical shifts of the β-carbon. On the other hand, although acrylamide was an inactive QR inducer and its predicted toxicity was relatively low, it showed toxicity under experimental conditions (Table 5). Biotransformation of acrylamide is thought to occur through glutathione conjugation and decarboxylation, with the formation of toxic glycinamide [36].

Induction of phase II enzymes and elevation of the glutathione level are well known to be involved in the toxic and carcinogenic effects of electrophiles and reactive forms of oxygen.

### 2.3. Interaction between DPPC Liposomes and (Meth)Acrylates

#### 2.3.1. NMR Chemical Shifts of Ha

Liposomes have been employed in model systems to study the interaction of lipid-soluble drugs and monomers with biological membranes [17,37–40]. Liposomes consist of lipid bilayers, and closely resemble the structure of biological membranes. Depending on their hydrophobicity, an exogenous hydrophobic compound will reside predominantly in liposomes and a hydrophilic one will be located in aqueous medium. NMR is one of the most powerful methods for studying not only the characterization of small unilamellar, large unilamellar and multilamellar liposomes [41,42] but also the interaction between (meth)acrylate monomers and liposomes as a model of cell membranes and transport phenomena across membranes [43,44]. We previously investigated the changes in ^1^H and ^13^C-NMR chemical shifts of methacrylates in DPPC liposomes. The chemical shift of Ha and β-carbon for methacrylates was shifted markedly to a higher field by their interaction with liposomes [38–40]. These findings may allow interpretation of the mechanism responsible for the hemolytic activity and cytotoxicity of monomers *in vitro* [39,40].

**Figure 2:**
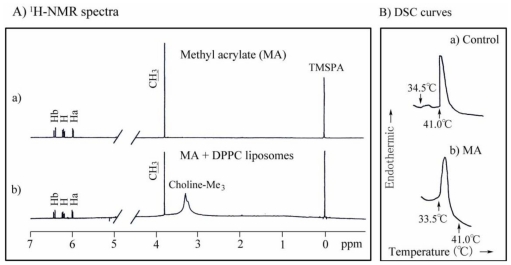
^1^H-NMR spectra (**A**) of: (a) MA and (b) DPPC liposome-bound MA (molar ratio: DPPC:MA = 10:1) in D_2_O (pD 7.0 phosphate buffer) at 25 °C, and DSC curves (**B**) of: (a) DPPC liposomes (control) and b) MA-treated DPPC liposomes (molar ratio: MA:DPPC = 1:1). The NMR chemical shift of Ha, Hb, H (α-CH) and CH_3_ was derived from MA molecule. By contrast, that of choline-Me_3_, *N*-(CH_3_)_3_ was derived from DPPC molecule [39,40].

We investigated the interaction between unilamellar DPPC liposomes and MMA, EMA, EA or MA using ^1^H-NMR spectroscopy. As an example, ^1^H-NMR spectra of MA and DPPC liposome-bound MA are shown in Figure 2.

The difference in chemical shift (ΔδHa) between monomers and liposomal membrane-bound monomers was calculated. As a typical example, the shift for each proton of the MMA molecule at 25 and 50 °C is shown in Table 6.

**Figure 3:**
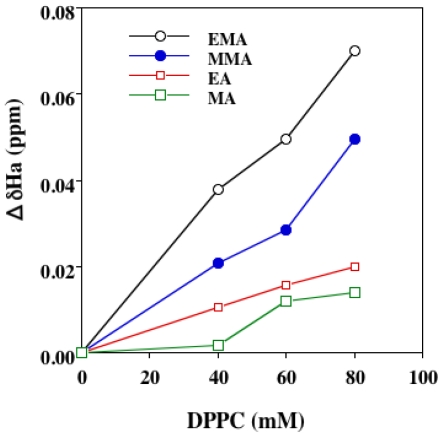
The chemical shift difference (ΔδHa, ppm) between free monomers and DPPC liposome-bound monomers as a function of DPPC concentration. The concentration of each monomer was 4 mM. The δ (ppm) values for Ha were determined with external TMSPA at 25 °C using ^1^H-NMR spectroscopy. The values represent the means of two or three separate experiments.

The ^1^H-NMR-chemical shift (ppm) of MMA in D_2_O (free monomer) for Ha, Hb, 2H and 5H was 5.72, 6.13, 1.94 and 3.79, respectively. That for Ha, Hb, 2H and 5H derived from membrane-bound monomers was determined by varying the DPPC liposomal concentrations. The ΔδHa for each proton in the MMA molecule at a DPPC:MMA molar ratio of 10:1 was investigated, and this revealed that the ΔδHa for Ha at 25 °C was about 10 times greater than that for the corresponding protons in the MMA molecule, the value being even higher at 50 °C. Since the DPPC concentration exceeds that of MMA monomers, Langmuir’s adsorption isotherm probably holds well. It was clear that the Ha attached to the β-carbon of monomers was highly impregnated into liposomes in a DPPC concentration-dependent manner. The relationship between ΔδHa for the monomers EMA, MMA, EA and MA and increasing DPPC concentration is shown in Figure 3. The decline in the ΔδHa value was dependent on DPPC concentration in the order EMA > MMA > EA > MA. The phase transition temperature (*T*) of DPPC liposomes was approximately 41 °C, and therefore the DPPC liposomes exist in a gel phase at 25 °C. We also examined the ΔδHa at 50 °C, at which DPPC liposomes exist in a liquid-crystalline phase. The ΔδHa at 50 °C declined in the order EMA > MMA > EA > MA, which was identical to that at 25 °C (data not shown). We also examined the QSAR between the ΔδHa at 40 mM DPPC and log 1/H_50_ for each monomer, and this revealed that the log 1/H_50_ value for monomers was linearly correlated with their ΔδHa (QSAR 11). This suggested that the hemolytic activity of monomers may be related to their membrane permeation activity.

#### 2.3.2. DSC Phase Transition Property

In earlier experiments, we examined changes in the phase transition properties of methacrylate-induced DPPC liposomes using DSC [38–40]. In this series, we investigated DSC changes in the phase transition temperature (*T*) (main transition peak), enthalpy (Δ*H*) and entropy (Δ*S*) of DPPC liposomes induced by (meth)acrylates. The results are shown in Table 7. The Δ*S* value was calculated according to Δ*S* = Δ*H*/*T* (Equation 4) and that has been is also given in Table 7. As an example of DSC scans, DSC curves of control and MA are also shown in Figure 2B. The *T*, Δ*H* and Δ*S* values for DPPC liposomes without any additives, as a control, were approximately 41.0 °C, 8.8 kcal/mol and 28.0 cal mol^−1^ K^−1^, respectively. Control showed two peaks of pre-transition at 34.5 °C with a very small Δ*H* and main transition at 41.0 °C with a large Δ*H*, 8.8 kcal/mol. Acrylates, MA, EA and nBA, showed a shift of *T* to a much lower temperature range of 32–34 °C, whereas methacrylates, MMA and EMA showed a shift of *T* to a slightly lower temperature range of 39.5–40.5 °C. The decrease in Δ*H* for nBA and EMA was greater than that for MA, EA and MMA. Shifts of the *T* and Δ*S* values for acrylates were greater than those for methacrylates, possibly due to differences between the α-CH_3_ and α-H substituents in the monomer molecule (Figure 1). The Δ*S* values for acrylates, MA and EA were greater than those for the corresponding methacrylates, MMA and EMA, possibly as a result of the effect of the steric factor of the α-substituent in the monomer molecule. The Δ*H* value for nBA was the lowest, probably in view of the hydrophobicity of its butyl substituent (Table 2).

MA and EA, although showing great *in vivo* toxicity, possessed less hemolytic activity (Table 1). Jain *et al.* [43] previously reported that antihemolytic small molecules have the ability to expand the lipid bilayer of biomembranes and cause large changes in the phase transition properties of the DPPC bilayer. Marique-Moreno *et al.* [44] investigated the effects of non-steroidal anti-inflammatory drug on human erythrocytes, and on liposomes as a cell membrane molecular model, and found that these drugs interacted strongly with dimyristoylphosphatidylcholine (DMPC) multilayers; DSC data also indicated a decrease in the melting (phase transition) temperature (*T*) of DMPC liposomes, which was attributed to destabilization of the gel phase. Taken together, it was concluded from these findings that the low hemolytic activity of MA, despite its high toxicity, may be attributable to its ability to expand the lipid bilayer of erythrocytes. The hemolytic activity of (meth)acrylates may be controlled by HF and hydrophobicity of the monomers, resulting from a good QSAR in the HF term and log *P*, whereas the *in vivo* toxicity of (meth)acrylates may be controlled by their π-electron density, σ-charge or *η* and *χ* reactivity principles, resulting from a good QSAR for these descriptors (Table 4). *In vivo* toxicity may be controlled by the Michael-type reactivity of the monomers [11]. As *in vivo* experiments are too complex to allow simple interpretation, liposome studies may help to clarify the mechanisms of *in vitro* and *in vivo* toxicity.

## 3. Experimental Section

### 3.1. Chemicals

The following chemicals and reagents were obtained from the indicated sources. Methyl acrylate (MA), ethyl acrylate (EA), methyl methacrylate (MMA), ethyl methacrylate (EMA) and *n*-butyl acrylate (nBA)(Tokyo Chemical Industry Co., Ltd., Tokyo, Japan); l-α-dipalmitoylphosphatidylcholine (DPPC)(Sigma Chemical Co., USA); deuterium oxide, 3-(trimethylsilyl)propionic acid sodium salt (TMSPA)(Merck, Darmstadt, Germany).

### 3.2. NMR Spectra

The ^1^H- and ^13^C-NMR chemical shift data for various monomers in chloroform-*d* (CDCl_3_) were taken from the literature [18,19]. Briefly, the chemical shifts of the indicated monomers were measured in CDCl_3_ at 35 °C at 125 and/or 500 MHz, respectively, using tetramethylsilane (TMS) as an internal standard.

### 3.3. NMR Study

Preparation of liposomes: Briefly, the method of DPPC liposome preparation was as follows: DPPC was accurately weighed and dissolved in chloroform. The solution was evaporated to a dry thin film on the bottom of a round-bottom test tube and left under vacuum for 30 min. Deuterium oxide (D_2_O, Merck, Darmstadt, Germany) in 0.01 M phosphate buffer at pD 7.0 was then added, and sonication was performed under a nitrogen atmosphere for 10 min at 60 °C. After incubation for 20 min at room temperature, unilamelar DPPC liposomes were prepared by centrifugation at 20,000× g for 15 min. ^1^H-NMR spectra were measured at 25 °C on a JEOL (Tokyo, Japan) JNM-GX 270 or ALPHA 500 instrument at a resolution of 0.01 ppm and 0.0013 ppm, respectively. An NMR sample tube with a coaxial capillary was used. The coaxial capillary with monomers was inserted into an NMR sample tube with the liposomes. Then, NMR spectra were measured at 25 °C and 50 °C, respectively. The external standard was TMSPA [17,38].

### 3.4. DSC Study

An aliquot sample (20 μL) of DPPC and the indicated concentration of monomer in 0.01 M phosphate buffer solution at pH 7.0 was placed into a DSC specimen container. The specimen was allowed to equilibrate for 14 h at 5 °C, then the specimen was scanned in a sealed calorimetric container on a DSC-Rigaku calorimeter (Rigaku Denki Co. Ltd., Tokyo, Japan) at a heating rate of 5 °C min^−1^ with a range setting of 0.5 mcal s^−1^. The instrument was calibrated with indium as a standard. The enthalpy (Δ*H*) was calculated from the area under the DSC curve [17,38,39].

### 3.5. Hemolytic Activity

The concentration eliciting 50% (H_50_) hemolysis of rabbit erythrocytes for (meth)acrylates was taken from the literature [3].

### 3.6. *In Vivo* Toxicity

LD_50_ (50% lethal dose) data for intraperitoneal injection of mice with acrylate and methacrylate monomers were taken from the literature [3]. Briefly, male albino ICR mice weighing 25 ± 5 g were used as the test animals, and the LD_50_ dose for each compound was calculated in terms of 7-day mortality.

### 3.7. Theoretical Parameters

Parameters *η*, *χ* and *ω* were calculated using Equations 1–3, respectively (Table 4). HOMO, LUMO, and heats of formation were taken from our reported studies [14,15]. Briefly, calculations of heats of formation were performed using the PM3/CONFLEX method. To obtain fine geometry details in the present study, initial geometry optimization was first performed using CONFLEX5 (Conflex, Tokyo, Japan). Thereafter, calculations using the PM3 method in the MOPAC 2000 program were carried out on a Tektronix CAChe workstation (Fujitsu Ltd., Japan).

## 4. Conclusions

QSARs between *in vitro* toxicity (1/H_50_) or *in vivo* toxicity (ipLD_50_) based on reported data and their NMR chemical shifts (δHa or δC_β_) or PM3-based theoretical parameters (HF, *η*, *χ*, *ω*) were investigated. There was a good linear QSAR between log 1/H_50_ and log *P* or HF for (meth)acrylates. Also, a good QSAR between ipLD_50_ and the δC_β_, *η*-, *χ*-, or *ω*-term for methacrylates was obtained, indicating that a common mechanism of action (Michael-type addition) of these monomers may be responsible for their *in vivo* toxicity. The interaction between DPPC liposomes and (meth)acrylates, investigated using NMR and DSC methods, indicated that the hemolytic activity of monomers may be controlled by their HF, which may be attributed to the monomer-induced phase transition properties of the erythrocyte lipid bilayer. NMR data may be an important tool for evaluating the biological activity of new vinyl monomers in medical and dental applications.

## Figures and Tables

**Table 1:** Hemolytic activity, *in vivo* toxicity and NMR chemical shifts for (meth)acrylates.

Compound	log 1/H_50_ (mole/L) a	7-days ipLD_50_ (mole/10^6^ g) a	δHa (ppm) b	δC_β_ (ppm) b
Methyl acrylate (MA)	0.63	2.95	5.82	130.56
Ethyl acrylate (EA)	0.95	5.98	5.807	130.24
*n*-Propyl acrylate (nPA)	1.59	5.80	5.809	130.22
*n*-Butyl acrylate (nBA)	2.61	6.64	5.805	130.21
Isobutyl acrylate (IBA)	2.84	5.92	5.813	130.23
Methyl methacrylate (MMA)	1.05	10.88	5.555	125.23
Ethyl methacrylate (EMA)	1.44	7.89	5.541	124.97
*n*-Propyl methacrylate (nPMA)	2.17	11.63	5.54	124.95
*n*-Butyl methacrylate (nBMA)	3.42	10.47	5.532	124.70

a Taken from Reference [3]; isopropyl acrylate and methacrylates, and *tert*-butyl acrylate have been omitted because no known NMR data are available for them;

b Taken from Reference [18].

**Table 2:** Physico-chemical parameters.

Com. a	Log *P*^b^	MR b	Vm (cm^2^/mole) b	*Q*^σ^ (C) c	Rate constant (*k*_app_) (liter mol^−1^min^−1^) d
MA	0.625	21.85	49.02	0.1666	52.0
EA	1.165	26.03	59.25	0.1662	26.6
nPA	1.705	26.5	69.47	--	--
nBA	2.245	31.15	79.70	0.1662	38.7
IBA	2.245	31.15	79.70	0.1662	--
MMA	0.945	27.5	59.25	0.1638	0.325
EMA	1.485	31.68	69.48	0.1634	0.139
nPMA	2.025	32.15	79.70	--	--
nBMA	2.565	36.8	89.94	0.1634	No appreciable rate

a For abbreviations see Table 1;

b Taken from Reference [3];

c Taken from Reference [1];

d Taken from Reference [20].

**Table 3:** Theoretical parameters.

Comp.	Heat of formation (HF) kcal/mol	*E*_HOMO_ eV	*η* eV	*χ* eV	*ω* eV
MA	−67.387	−11.066	5.492	5.574	2.829
EA	−72.173	−11.040	5.495	5.546	2.799
nPA	−77.404	−11.044	5.495	5.550	2.803
nBA	−82.791	−11.045	5.495	5.550	2.803
IBA	−82.435	−11.042	5.495	5.548	2.801
MMA	−74.768	−10.548	5.245	5.303	2.681
EMA	−79.542	−10.524	5.249	5.278	2.654
nPMA	−84.767	−10.529	5.248	5.281	2.657
nBMA	−90.156	−10.530	5.248	5.282	2.658

Values were taken from References [14,15].

**Table 4:** Quantitative structure-property relationship (QSPR) (**A**) and quantitative structure-activity relationship (QSAR) (**B**) for (meth)acrylates.

(**A**)	

$\text{Chemical hardness}:\eta =(E_{\text{LUMO}}-E_{\text{HOMO}})/2$	Equation (1)
$\text{Electronegativity}:\chi =-(E_{\text{LUMO}}+E_{\text{HOMO}})/2$	Equation (2)
$\text{Electrophilicity}:\omega =\chi^{2}/2\eta$	Equation (3)
Gibbs free energy:ΔG=ΔH-TΔS	Equation (4)
For (meth)acrylates:	
*k*_app_ = −941.33 (±9.43) + 7.53 (±1.64) δC_β_ (*n* = 5, *r*^2^ = 0.88, *p* < 0.05)	QSPR 1
For MA, EA, MMA and EMA at 40 mM DPPC:	
ΔδHa = −0.320 (±0.012) − 0.005 (±0.001) HF (*n* = 4, *r*^2^ = 0.92, *p* < 0.05)	QSPR 2

(**B**)	

For (meth)acrylates:	
Log 1/H_50_ = −0.44 (±0.24) − 0.36 (±0.12) log *P* (*n* = 9, *r*^2^ = 0.95, *p* < 0.001)	QSAR 1
Log 1/H_50_ = −5.55 (±0.27) − 0.09 (±0.14) HF (*n* = 9, *r*^2^ = 0.86, *p* < 0.001)	QSAR 2
ipLD_50_ = 123.0 (±1.5) − 0.9 (±0.2) δC_β_ (*n* = 9, *r*^2^ = 0.78, *p* < 0.01)	QSAR 3
ipLD_50_ = 109.0 (±1.5) − 17.8 (±3.6) δHa (*n* = 9, *r*^2^ = 0.78, *p* < 0.01)	QSAR 4
ipLD_50_ = 1.02 (±0.26) − 0.01 (±0.03) δC_β_ + 1.40(±0.14) log *P* (*n* = 9, *r*^2^ = 0.94, *p* < 0.001)	QSAR 5
ipLD_50_ = −1.1 + 8.8 (±2.0) log *P* − 2.1 (±0.6) log *P*^2^ (*n* = 9, *r*^2^ = 0.78, *p* < 0.01)	QSAR 6
ipLD_50_ = 270.2 (±16) − 1592.8 (±429.9) *Q*^σ^(C) (n = 7, *r*^2^ = 0.73, *p* < 0.05)	QSAR 7
ipLD_50_ = 111.2 (±1.5) − 19.3 (±4.2) *η* (*n* = 9, *r*^2^ = 0.75, *p* < 0.01)	QSAR 8
ipLD_50_ = 105.1 (±1.5) − 17.5 (±3.7) *χ* (*n* = 9, *r*^2^ = 0.75, *p* < 0.01)	QSAR 9
ipLD50 = 98.5 (±1.4) − 33.1 (±6.6) *ω* (*n* = 9, *r*^2^ = 0.78, *p* < 0.01)	QSAR 10
For MA, EA, MMA and EMA:	
Log 1/H_50_ = 0.57 (±0.13) + 117.55 (±27.48) ΔδHa (*n* = 4, *r*^2^ = 0.90, *p* < 0.05)	QSAR 11

**Table 5:** Concentration of double quinone reductase (QR) in Hepa 1clc7 cells, glutathione reactivity (*k*_app_), *in vivo* oral or ipLD_50_ (mouse) and NMR chemical shifts for reactive acrylates.

Name	Acrylate	Concentration of QR	*k*_app_	Reported oral-LD_50_, (mg kg^−1^)	NMR chemical shift h

	Structure	(mM) a	(M^−1^min^−1^) b	(ipLD_50_, mol kg^−1^) c	δHa(δC_β_), ppm
MA	CH_2_=CHCOOCH_3_	20	41.8	857 (5.5) d	5.825(130.56)
MMA	CH_2_=C(CH_3_)COOCH_3_	I	16.8	5,197 (10.3) d	5.555(125.23)
Acrolein	CH_2_=CHCHO	130	94.6	40 (0.5) e	6.495(137.57)
Acrylonitrile	CH_2_=CHC≡N	50	91.4	27 (0.8) f	6.083(137.14)
Acrylamide	CH_2_=CHCONH_2_	I	17.9	107 (8.4) g	5.700(127.38)

a Taken from Talalay *et al*. [25]. I, inactive, <20% increase in specific activity at 200 mM;

b Calculated using the QSPR 1;

c Calculated using the QSAR 3;

d Taken from Reference [2];

e Taken from Reference [26];

f Taken from Reference [27];

g Taken from Reference [28];

h Taken from Hatada *et al.* [18].

**Table 6:** The chemical shift difference (ΔδHa, ppm) between free MMA and DPPC liposome-bound MMA at 25 and 50 °C.

MMA, structure and numbering	H attached to the carbon	ΔδHa, ppm	

		25 °C	50 °C
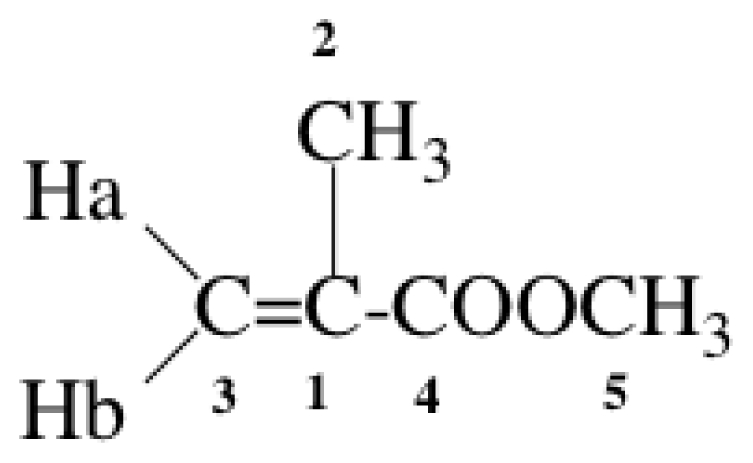	Ha	−0.01	−0.05
	Hb	−0.005	−0.01
	5H	−0.004	0.00
	2H	−0.001	0.03

MMA, 4 mM; DPPC:MMA = 10:1 (molar ratio). The negative value for each proton in the MMA molecule exhibited a shift to a higher field, whereas the corresponding positive value exhibited a shift to a lower field.

**Table 7:** Changes in DSC phase transition properties of multilamellar DPPC liposomes induced by (meth)acrylates.

Compound	Phase transition temperature (*T*) °C	Enthalpy (Δ*H*) kcal/mol	Entropy (Δ*E*) cal mo1^−1^K^−1^
Control	41.0	8.8	28.03
MA a	33.5	7.9	25.77
MMA a	39.5	6.7	21.44
Control	41.5	8.9	28.30
EA b	32.5	7.7	25.20
nBA b	31.5	4.0	13.13
EMA b	40.5	5.7	18.18

Values are the means for two or three separate experiments. *T*: S.E. < 0.01%; Δ*H*: S.E. < 10%.; 75 mM DPPC.

a 75 mM;

b 25 mM.
